# Society Meetings

**Published:** 1910-04

**Authors:** 


### SOCIETY MEETINGS.

---

The Buffalo Academy of Medicine held meetings during the month of March, 1910, as follows:

*Section on Surgery.*—Tuesday evening, March 1. Program: Intestinal Diverticula; their Pathology and Clinical Significance, John A. Hartwell, of New York.

*Section on Medicine.*—Tuesday evening, March 8. Program: Demonstration of Valve Motions of Heart, using water as a circulating medium, with electrical interior illumination, John W. Radu; Lobar Pneumonia in Children, Allen M. Baines, Professor of Diseases of Children of the University of Toronto, Canada; The use of the $x$-Ray in Early

Diagnosis of Chronic Joint Lesions. Illustrated with lantern slides, R. O. Meisenbach.

*Section on Obstetrics.*—Tuesday evening, March 15. Program: (a) A Study of Uterine Myomata with Reference to an Explanation of their Symptoms, John A. Sampson, Albany, N. Y.; (b) The Present Status of the Serum Treatment of Cancer, Harvey R. Gaylord.

*Section on Pathology.*—Tuesday evening, March 22. Program: Study on Metabolism, R. H. Chittenden, New Haven, Conn.

---

The Rochester Academy of Medicine held meetings during the month of March, 1910, at the Hotel Seneca, as follows:

*Section on General Medicine.*—Wednesday evening, March 2. Program: Serum Therapy, Professor E. R. Larned, Buffalo.

*Section on Surgery.*—Wednesday evening, March 9. Program: Bow Legs, L. A. Whitney.

*Section on Obstetrics.*—Wednesday evening, March 16. Program: Cesarean Section, abdominal and vaginal, and indications for each, W. B. Jones; Bow Legs, L. A. Whitney.

*Section on Public Health.*—Wednesday evening, March 23. Program: The Eye Troubles of School Children, Leonard W. Jones.

---

The Tenth Anniversary of Groszmann School will be observed Saturday, April 2, and a further program April 21 and 22, 1910. In commemoration of the tenth anniversary of the Groszmann School on April 1, the annual tree planting will be conducted, and on the following day, April 2, the teachers and pupils will take part in special exercises.

On April 21 and 22, there will be a conference in connection with the anniversary celebration, held under the auspices of the National Association for the Study and Education of Exceptional Children in the assembly hall of the New York University School of Pedagogy, Washington Square, New York. Prominent men and women from all parts of the United States will be in attendance. The general topic will be "Exceptional Children," and the discussion will tend to a wider and better understanding of the needs of that class of children.

The celebration committee consists of Dr. George Alexander Kohut, chairman; Dr. Simon P. Goodhart and W. H. Groszmann. Dr. Thomas M. Balliett, dean of the School of Pedagogy, New York, will preside over the conference during which a number of leading experts in medicine and education will take part. All who are interested in this grave problem which is not merely of educational, but preëminently of sociological significance, are invited to attend. It is planned to hold such conferences annually.

---

THE National Confederation of State Medical Examining and Licensing Boards will hold its twentieth annual meeting at the Southern Hotel. St. Louis, June 6, 1910, at 10.00 a. m., under the presidency of Dr. A. Ravogli, of Cincinnati. The secretary is Dr. Murray Galt Motter of Washington, D. C.

The confederation has decided to request some of the leading clinicians connected with medical schools to prepare and read papers at this meeting, regarding the means whereby it may be possible to give more practical clinical work to medical students, with the view to enable them to meet a practical examination before state medical examining boards. An important contribution has been promised from the Carnegie Foundation, which has been investigating medical education in this country. A general invitation is extended to all who are interested in improving medical education.

